# An ingenious 3D-printed navigating template for accurate screw placement in lumbar spinal stenosis

**DOI:** 10.3389/fsurg.2025.1635255

**Published:** 2025-09-08

**Authors:** Yaoshen Zhang, Kangpeng Li, Rui Ma, Yuzeng Liu, Qiang Zhang, Yong Hai

**Affiliations:** ^1^ Department of Orthopedics, Beijing Chaoyang Hospital, Capital Medical University, Beijing, China; ^2^ Department of Orthopedics, Beijing Ditan Hospital, Capital Medical University, Beijing, China

**Keywords:** 3D printing, lumbar spinal stenosis, TLIF, navigating template, spinal fusion

## Abstract

**Objective:**

To investigate the feasibility, accuracy, and safety of using a 3D-printed navigating template in transforaminal lumbar intervertebral fusion (TLIF) for treating lumbar spinal stenosis (LSS).

**Methods:**

A retrospective analysis was conducted on single-segment lumbar vertebrae treated with TILF at Beijing Ditan Hospital between May 2023 and May 2024. Clinical data were collected from patients diagnosed with LSS. Among them, 36 patients who underwent surgery using a 3D-printed navigating template were assigned to the template group, while another 36 patients with similar baseline characteristics were included in the control group. The following parameters were recorded: operative time, blood loss, frequency and duration of fluoroscopy, accuracy of screw placement, and incidence of complications related to spinal cord or nerve injuries.

**Results:**

The operation time and blood loss in the template group were significantly lower than those in the control group (*P* < 0.05). The fluoroscopy time and frequency on the template side were also significantly lower than those on the puncture side and in the control group (*P* < 0.05). Notably, there were no grade 2 screws observed in the template group, whereas 14 grade 2 screws were identified in the control group. Furthermore, the proportion of grade 0 screws on both sides in the template group was significantly higher compared to the control group (*P* < 0.05). However, no statistically significant difference was observed between the two sides within the template group (*P* > 0.05). Additionally, none of the patients experienced complications such as spinal cord or nerve injury.

**Conclusion:**

The application of 3D-printed navigating templates in the treatment of LSS using TLIF is feasible. Despite the need to account for potential inaccuracies caused by skin movement and changes in body position during surgery, this technique represents a novel and viable minimally invasive approach for screw placement.

## Introduction

Lumbar spinal stenosis (LSS) is a prevalent cause of low back pain and leg pain among middle-aged and elderly people. Approximately 8%–11% of individuals aged 65 and older experience symptomatic LSS (1). This condition can result in chronic pain, impair walking ability, significantly compromise quality of life, and lead to increased medical costs (2). With the increasing adoption of minimally invasive concepts in spinal surgery, minimally invasive spinal techniques have achieved remarkable advancements in treating LSS (3–5). For patients suffering from lumbar instability or spondylolisthesis, minimally invasive fusion not only offers enhanced stability and reduced surgical trauma but also demonstrates advantages such as minimal blood loss and accelerated recovery compared to traditional open surgery (6). Pedicle screws are crucial for reconstructing the three-column stability of the spine and are extensively utilized in treating conditions such as LSS. Inaccurate placement of pedicle screws may lead to damage of vital anatomical structures, including adjacent nerves, blood vessels, and the spinal cord, potentially causing irreversible consequences (7). Most patients with LSS are elderly individuals who typically present with lumbar degeneration, bone hyperplasia, and anatomical structural disorders. These conditions necessitate that surgeons possess extensive clinical experience and rely on repeated intraoperative fluoroscopy to ensure precision. Earlier studies reported that the incidence of pedicle perforation ranged from 1.5% to 58.0% (8–10). Therefore, enhancing the accuracy of screw placement through the integration of novel technologies is essential.

3D printing, also referred to as rapid prototyping, involves designing, planning, and simulating objects using computer-aided design software (11). The resulting designs are then input into a 3D printer, which manufactures the object layer by layer according to the design specifications. These layers are subsequently stacked to produce a precise 1:1 physical model (12). This technology was initially applied in the medical field, specifically in dentistry and maxillofacial surger (13–15). In recent years, its application in the field of orthopedics has grown significantly, encompassing various aspects such as preoperative planning, personalized implants, surgical templates, and biological bone scaffold (16–19). This has notably enhanced both the precision of surgical procedures and patient prognoses. Its advantages are evident: precise preoperative planning, individualized intraoperative fluoroscopy, reduced operation time, and improved accuracy. In practical applications, we anticipate that personalized 3D-printed navigation templates, designed based on patients' anatomical data, will assist surgeons in performing accurate osteotomies, drilling, or screw placements while minimizing the risk of nerve injury.

In this study, an innovatively designed 3D-printed navigating template was applied to patients with LSS for percutaneous pedicle screw placement. Between May 2023 and May 2024, a total of 36 patients with LSS were treated using this technology. The following parameters were evaluated: operation time, blood loss, fluoroscopy duration and frequency, accuracy of screw placement, and complications related to spinal cord and nerve injury.

## Methods

### Ethical statement

We confirm that Helsinki Declaration has been followed for involving human subjects in this study. This study protocol has been reviewed and approved by the Ethics Committee of Beijing Ditan Hospital, Capital Medical University. Written informed consent has been obtained from all the patients and their anonymous information will be published in this article. All methods were performed in accordance with the relevant guidelines and regulations.

### General information

From May 2023 to May 2024, patients with single-segment LSS who underwent TLIF in our department were enrolled. All patients underwent standard lumbar anteroposterior and lateral x-rays, flexion-extension dynamic x-rays, lumbar CT, magnetic resonance imaging (MRI), and bone mineral density assessments. Patients with other diseases, such as vertebral infections or tumors, were excluded from the study. Thirty-six patients who utilized 3D-printed navigating templates during surgery were assigned to the template group, while 36 patients with comparable baseline characteristics were allocated to the control group. All patients exhibited symptoms of low back pain, unilateral lower limb pain and/or numbness, intermittent claudication, and dynamic x-ray shows instability of the affected lumbar segment, with specific signs including horizontal displacement between vertebral bodies (but not progressed to lumbar spondylolisthesis), asymmetry and widening of facet joints, and narrowing of intervertebral space height, etc. Demographic data, including gender, age, and surgical segment, were meticulously recorded.

### Template making

**Figure 1:**
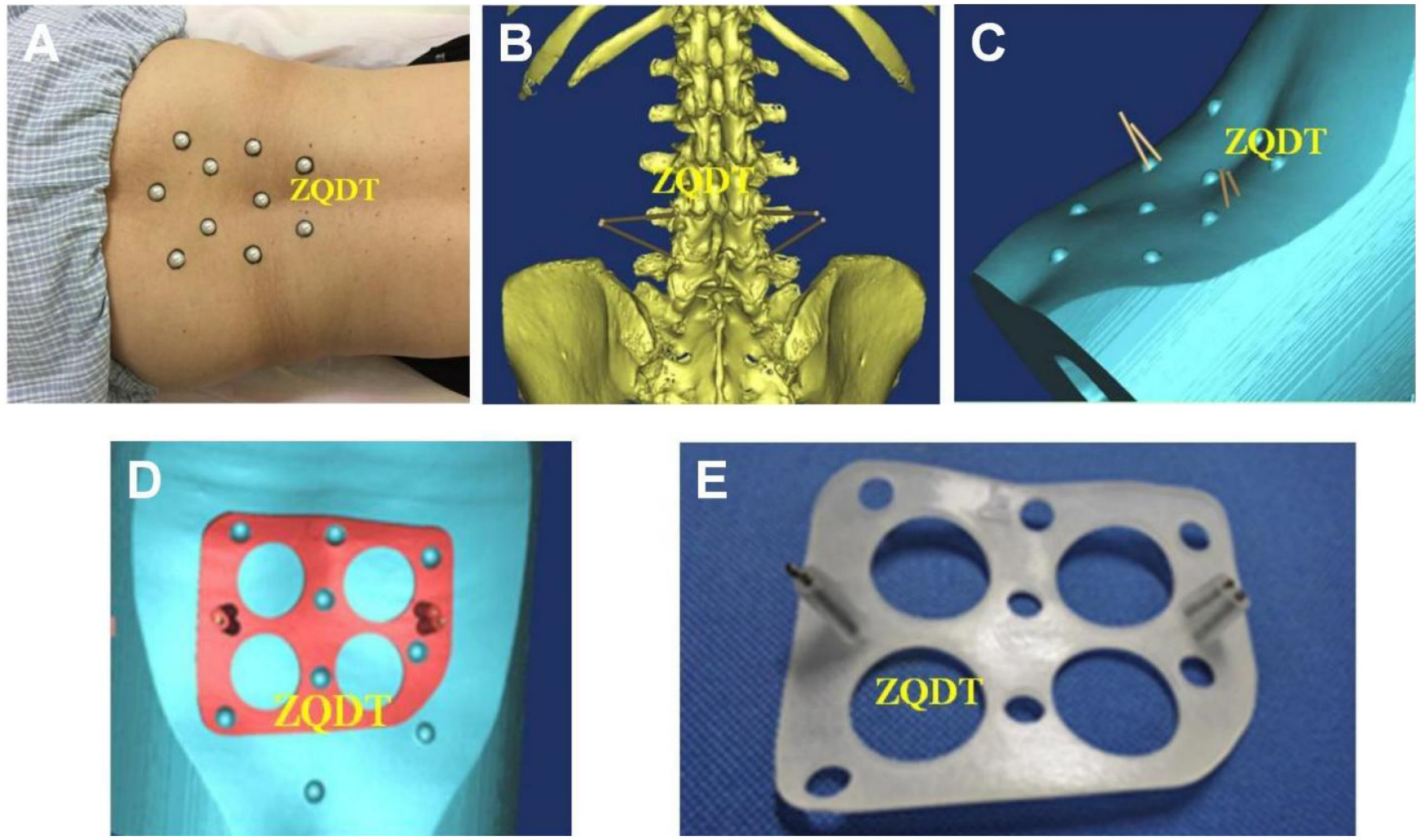
**(A)** The positioning markers were selected on the skin around the surgical segment of the patient, and the marks were made on the skin. The markers could be attached to the skin and visualized in CT scanning. Spine CT with slice thickness of 1 mm was performed in the prone position. **(B,C)** The original CT DICOM data were collected and input into the computer, and the computer aided design (CAD) software Mimics10.01 was used for digital three-dimensional reconstruction, and the operation plan and simulation were carried out to find the best position of screw placement, and the direction and depth of virtual needle insertion were set. **(D)** The reverse engineering software Geomagic Studio7 was used to design a navigation template with a guide channel that was closely attached to the skin. The reconstruction results were input into the 3D printer, and the real model of the percutaneous guide plate was made according to the design drawings. **(E)** The design data were imported into the 3D printer in STL format to produce a real model of the percutaneous guide plate.

Landmarks were identified and marked on the skin surrounding the surgical segment. Adhesive markers were affixed to the skin and were clearly visualized during CT scanning. The patient was positioned in a prone posture for CT acquisition with a slice thickness of 1 mm. The original CT dicom data were collected, transferred to the computer, and imported into CAD Mimics 10.01 software for digital 3D reconstruction. Subsequently, surgical planning and simulation were performed to determine the optimal screw placement, and the virtual trajectory and depth of the needle were established. Thereafter, Geomagic Studio 7 engineering software was utilized to design a navigating template featuring a guide channel that conformed precisely to the skin surface. Finally, the reconstructed data were exported to a 3D printer, and the template model was fabricated (Figure 1).

### Preoperative management

All patients were evaluated by at least two experienced spine surgeons, one of whom held a senior professional title. The surgical plan was formulated and finalized by senior spine surgeons. For patients with hypertension, diabetes, or other systemic diseases, consultations with relevant departments were conducted for treatment, and contraindications were ruled out prior to surgery. The length of the pedicle screws was measured based on preoperative axial CT images of the lumbar pedicle plane. The final screw length of all patients was satisfactory in accordance with the ideal preoperative plan.

### Surgical methods

Patients in both groups were positioned prone with a suspended abdomen under general anesthesia. Preoperative fluoroscopy was utilized to determine the surface projection of the pedicle of the affected segment and establish landmarks. In the template group, template navigation was employed for screw placement on the side of the lower limb without clinical manifestations (referred to as the template side), while the Wiltse approach was used for screw placement on the side with clinical manifestations (referred to as the puncture side). The 3D-printed template and skin markers were sterilized by low temperature plasma before operation. The operating bed was routinely disinfected and covered with towels. The skin markers were placed on the skin landmarks to locate the position of the template, so that the navigation template was closely fitted to the skin. The Kirschner wire was inserted into the template side through the guiding channel on the template, and the position of the Kirschner wire was confirmed by fluoroscopy. On the puncture side, the skin, subcutaneous tissue and lumbodorsal fascia were cut in sequence, and the interspace between the multifidus muscle and the longissimus muscle was used to expose the superior and inferior facet joints and lamina. The inferior facet joint of the upper vertebral body, the superior facet joint of the lower vertebral body and the lateral lamina of the upper and lower lamina were removed with bone knife and laminectomy forceps. The herniated disc, nerve root and dural sac were exposed, the nerve root canal was explored, and fully decompressed. The nerve root and dural sac were pulled medially to protect the superior nerve root. The annulus fibrosus was incised and the protruding nucleus pulposus tissue was removed. The intervertebral disc and cartilage endplate were cleaned up to the bony endplate of the upper and lower vertebral bodies with different types of disc retractor and curette. Decompression of the spinal canal and nerve root canal was completed. The autogenous bone block or allogeneic bone block under decompression bite was trimmed and inserted into the intervertebral space, and a single Cage filled with bone particles was placed oblique into the intervertebral space. On the puncture side, pedicle puncture was performed according to the anatomical landmarks, and the puncture point and puncture direction were adjusted by fluoroscopy. After the positioning was good, the guide needle was placed, and the pedicle screw was selected and inserted according to the length measured before operation. Longitudinal connecting rods were placed under direct vision on the puncture side and percutaneously on the template side. Both sides were pressurized and fixed by tightening the tail nut of the pedicle screw. After operation, a drainage tube was placed on the puncture side and the incision was sutured.

**Figure 2:**
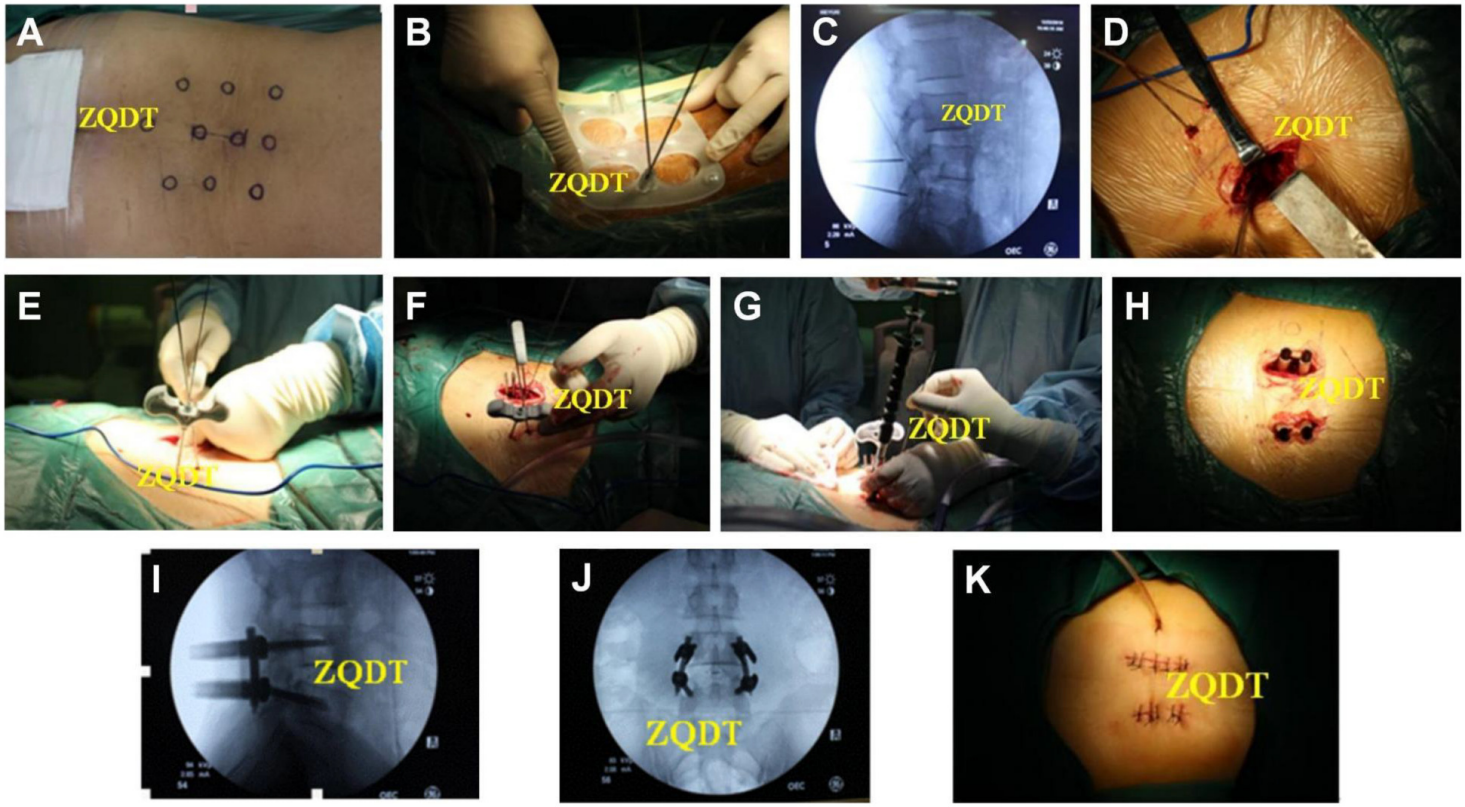
**(A)** Intraoperative position and body surface markers. **(B)** Markers were placed according to the markers, guides were placed according to the markers, and Kirschner wires were implanted under template navigation. **(C)** The position of Kirschner wire was determined by fluoroscopy. **(D)** For patients with unilateral symptoms, TLIF was performed by percutaneous pedicle screw placement on one side, and TLIF was performed by Wiltse approach on the other side. **(E)** Kirschner wires were punctured and a guide needle was placed. **(F)** Pedicle screw placement was completed along the guide needle. **(G)** The longitudinal connecting rod was placed under direct vision on the incised side. **(H)** A longitudinal connecting rod was percutaneously placed on the template side. **(I,J)** Fluoroscopy showed that the internal fixation was in good position. **(K)** A drainage tube was placed on the incision side of the paravertebral approach.

In the control group, the skin, subcutaneous tissue and lumbar dorsal fascia were cut in turn, the paraspinal muscles were separated along the spinous process, and the lamina and facet joint of the diseased segment were exposed. Pedicle screws were placed by free hand at the bilateral pedicle of the upper and lower vertebral bodies of the lesion. During the operation, the fluoroscopy equipment needed to be used for initial positioning to determine the entry point of the screw, and then to confirm the direction of the guide pin in the anteroposterior position, and to monitor the depth of the guide pin in the lateral position in real time, and then to confirm the position and length of all the screws by alternating anteroposterior and lateral fluoroscopy. Decompression of spinal canal and nerve root canal was performed in the same group (Figure 2).

### Indicators of observation

The operation time, blood loss, fluoroscopy time and frequency, as well as spinal cord and nerve injury complications were recorded. In the test group, the fluoroscopy time (Total exposure time to radiation exposure) and frequency (The number of times the C-arm x-ray machine was started for fluoroscopy) were separately observed on the puncture side and the template side. Postoperative sagittal and axial CT images were used to evaluate the relationship between the pedicle screw and the pedicle cortex, thereby assessing the accuracy of screw placement. The grading criteria for screw placement accuracy were as follows: Grade 0, the screw was entirely within the pedicle; Grade 1, screw penetration ≤2 mm; Grade 2, screw penetration >2 mm and <4 mm; Grade 3, the screw breached the cortex by ≥4 mm (20).

### Statistical analysis

SPSS 13.0 software was utilized for data analysis. The operation time and blood loss between the trial group and the control group were compared using the independent-sample Wilcoxon rank-sum test. The fluoroscopy time between the template side and the puncture side was compared using the paired-sample Wilcoxon signed-rank test. Fisher's exact test was employed to compare the accuracy of pedicle screw placement between groups. A *p*-value less than 0.05 was considered statistically significant.

## Results

A total of 72 patients were enrolled, with 36 assigned to the template group and 36 to the control group. The male-to-female ratio was 1:1 in both groups. The mean age in the template group was 65.7 years, with a disease duration of 23.9 months. In the control group, the mean age was 66.8 years, and the disease duration was 22.1 months. The mean BMI was 22.45 in the template group and 22.87 in the control group. The most frequently affected segment was L4/L5 (25 cases), followed by L5/S1 (11 cases) in the template group, while in the control group, these segments involved 21 and 15 cases, respectively. No statistically significant differences were observed concerning sex, age, BMI, disease duration, or affected spinal segment.

A total of 144 pedicle screws were placed in each group, with 72 screws inserted on the template side and the puncture side in the trial group. The operation time, blood loss, fluoroscopy time, fluoroscopy frequency, and accuracy of screw placement are summarized in Table 1. The operation time and blood loss in the template group were significantly lower than those in the control group (*P* < 0.05). In the template group, the fluoroscopy time and frequency on the template side were significantly lower than those on the puncture side and in the control group (*P* < 0.05). No significant difference was observed between the puncture side and the control group (*P* > 0.05). There were no grade 2 screws in the trial group, whereas 4 grade 2 screws were identified in the control group. The proportion of grade 0 screws in the trial group was significantly higher than that in the control group on both sides (*P* < 0.05). No significant difference was found between the two sides of the template group (*P* > 0.05). No spinal cord or nerve injuries occurred in any of the patients.

**Table 1:** Intraoperative observation indicators.

Group	*n*	Operation time/min (mean, 95% CI)	Blood loss/ml (mean)	Perspective time/s (mean)	Perspective frequency (median)	Accuracy of pedicle screw placement		
						Grade 0	Grade 1	Grade 2
Template side	72	133 [125.36, 140.64]*	189 [174.91, 203.09]*	0.96 [0.80, 1.10]*^,^**	1 [1.28, 1.77]*^,^**	64*	8	0
Puncture side	72			5.60 [5.00, 6.20]	7 [6.30, 7.82]	60	12	0
Control	144	169 [161.70, 177.02]	328 [304.40, 351.60]	5.92 [5.35, 6.55]	7 [6.27, 7.73]	96	34	14

* *P* < 0.05, compared with control group.

** *P* < 0.05, compared with puncture side.

## Discussion

In this study, based on the CT data, individualized designs were created with computer assistance, and 3D printing technology was utilized to fabricate the templates. The operation could achieve screw placement under template navigation, unaffected by abnormal anatomical structures caused by bone hyperplasia, thereby enhancing the accuracy of screw placement and reducing complications. In the template group, all screws on the template side were graded as either 0 or 1, with grade 0 screws accounting for about 90%, clearly demonstrating the high precision of navigating templates. Additionally, the results indicated that although manual techniques were used for screw placement on the puncture side of the template group and control group, the accuracy of screw placement on the puncture side of the template group was higher than that of the control group, with no significant difference between the puncture side and the template side. This may be attributed to the insertion of a guide needle after puncture on the puncture side, followed by screw insertion along the guide needle, ensuring the same screw position as achieved under fluoroscopy. In contrast, in the control group, despite good positioning needle placement under fluoroscopy, the screw position became abnormal after the positioning needle was withdrawn. These findings suggest that inserting a guide needle after manual puncture and subsequently inserting the screw along the guide needle can also improve the accuracy of screw placement. Also, This discrepancy may be attributed to the Wiltse approach on the puncture side in the trial group and the different approach in the control group.

In this study, there were no occurrences of grade 3 screw malpositioning, spinal cord injury, nerve damage, or other complications, as all procedures were conducted by experienced senior spine surgeons. The learning curve for traditional manual pedicle screw placement is steep and challenging, demanding that surgeons possess a deep familiarity with the surgical technique and proces. It takes young surgeons a considerable amount of time to fully master this skill. With template-based navigation, *Mimics* software can be utilized for preoperative individualized 3D reconstruction of the lumbar spine, enabling precise surgical planning and simulation to determine the optimal position for screw placement, as well as setting the direction and depth of virtual needle insertion. This process is straightforward and can be acquired through short-term training. The design of virtual surgery offers inexperienced young surgeons an opportunity for preoperative practice, allowing them to comprehend surrounding anatomical structures and gain proficiency in surgical techniques. This not only reduces the complexity of the surgery and the risk of complications but also enhances the efficiency and success rate of actual operations.

Traditional free-hand screw placement involves repeated fluoroscopy for adjusting the puncture point and direction, thereby increasing the frequency and duration of fluoroscopy, prolonging operation time, and causing greater blood loss. The results of this study demonstrated that both the fluoroscopy time and frequency in the template-guided group were significantly lower than those in the manual puncture group and the control group. This indicates that template-based navigation can effectively reduce fluoroscopy time and frequency. In addition, if fluoroscopy reveals an unsatisfactory screw position following manual screw placement, it is often necessary to adjust the screw direction, which may lead to screw loosening. Template navigation transforms fluoroscopy-guided screw placement into fluoroscopy-verified screw placement, allowing the procedure to be completed after one or two fluoroscopy checks. In this study, 80% of patients required only a single fluoroscopy to complete the operation, thereby reducing both the number of fluoroscopies and the overall operation time.

Traditional open TLIF for patients with LSS involves dissection of the paraspinal muscles, which may lead to denervation, lower back discomfort, paraspinal tissue injury, and decreased lumbar stability. However, there exists a natural anatomical gap between the multifidus muscle and the longissimus muscle. The Wiltse approach utilizes this gap to access the operative area without extensive muscle dissection. Through an expanded channel, the upper and lower articular processes, lamina, and pedicle screw entry points of the affected intervertebral space can be reached. In cases where an expanded channel is unavailable, the method described in this study can still be employed by entering through the gap between the multifidus and longissimus muscles. A retractor can then be used to complete the procedure. Due to the mobility of the skin, pedicle screw placement can be achieved by gently pulling the skin at the incision site, resulting in a smaller surgical incision. In this study, the paravertebral muscles were not dissected, and pedicle screws were placed percutaneously on the template side. Decompression surgery via the Wiltse approach on the puncture side demonstrates less trauma, shorter operation time, reduced blood loss, and comparable outcomes in terms of decompression, fusion, and internal fixation compared to traditional open surgery. Cawley et al. conducted ultrasonic muscle quantitative measurements and electromyography studies on the multifidus muscle during lumbar surgery and found that the Wiltse approach better preserves the innervation of the multifidus muscle, reduces the occurrence of muscle atrophy, and significantly decreases the incidence of postoperative lower back discomfort (21).

In this study, skin movement and changes in the patient's body position were potential sources of error, and their impact on the surgery included: Amplified Skin Movement: The thickness and compliance of subcutaneous adipose tissue in obese patients significantly increase the potential for skin shift between the preoperative CT scan (used for template design) and the intraoperative position. Even subtle patient repositioning or respiratory movements can cause greater displacement of the skin surface relative to the underlying bony anatomy compared to non-obese patients. This displacement directly translates to misalignment of the template's guide channels relative to the intended entry points and trajectories on the bone; Impaired Template-Skin Contact and Stability: Achieving a stable and precise fit of the template onto the skin surface is crucial. In obese patients, the irregular and softer contours of the back, potential skin folds, and difficulty in achieving firm pressure can compromise the template's conformity and stability. This may lead to template wobble or partial lifting during drilling, introducing deviation; Challenges in Landmark Palpation/Registration: While our template aims to minimize reliance on manual palpation, initial positioning often still references palpable bony landmarks (e.g., spinous processes). Obesity can make these landmarks difficult or impossible to palpate reliably, potentially affecting the initial placement accuracy of the template before any drilling begins. Furthermore, the registration between the virtual template and the patient's anatomy during planning might be less precise if skin surface models derived from CT are significantly deformed by adipose tissue compression in different positions. Meticulous Patient Positioning and Skin Preparation: Ensuring consistent patient positioning (prone on a radiolucent frame) between CT and surgery, minimizing repositioning during draping, and using adhesive dressings or tapes to stabilize the skin around the template application site might help reduce skin shift.

The solutions include: Enhanced Template Design Considerations: Future iterations could explore designs with broader bases, contoured padding, or integrated stabilization features (e.g., light vacuum suction—though sterility is a concern) to improve conformity and stability on irregular surfaces. Utilizing multiple smaller, potentially interconnected templates over specific segments might also improve local stability; Intraoperative Verification: Emphasizing the critical importance of intraoperative fluoroscopy (AP and lateral views) before drilling commences and after guidewire placement becomes even more paramount in obese patients. This allows for the detection and correction of any significant misalignment introduced by skin/template shift; Patient Selection: We explicitly state that severe obesity (e.g., BMI > 35–40 kg/m^2^) may currently represent a relative contraindication or a significant risk factor for reduced accuracy with this technique, and surgeon discretion is advised. The technique may be most reliable in patients with low to moderate BMI where skin shift is less pronounced.

The computer-aided navigation system employs infrared or electromagnetic technology to achieve precise placement of lumbar pedicle screws during surger (22). However, this system has several limitations, including high equipment costs, complex operation, a steep learning curve, and prolonged surgical time, which restrict its widespread clinical application. In contrast, the application of 3D-printed templates is simple, convenient, and allows for individualized design. Nevertheless, internal template navigation in spinal surgery may lead to inaccurate pedicle screw placement due to vertebral body movement during the procedure (23). Similarly, skin mobility significantly affects the accuracy of 3D-printed template navigation. To address this issue, our study proposes selecting positioning landmarks on the skin surrounding the surgical segment prior to CT scanning. These landmarks should be located at superficial bony sites, particularly palpable anatomical landmarks such as spinous processes and bilateral upper and lower joint processes. Markers are then placed on the skin, adhering firmly to it and visible during CT scanning. During surgery, these markers are repositioned in the same locations as before. All patients are positioned on a postural fixation frame prior to CT scanning to ensure consistency between the scanning position and the surgical screw placement position. The results demonstrate that this method effectively mitigates the impact of skin mobility on template navigation accuracy. Also, since the radiation exposure data were only retained on the C-arm instrument for one year, we unfortunately did not have the means to quantify the radiation exposure dose in mGy. Finally, it is indeed difficult to identify bony markers in obese patients, and our template was not tested for use in obese patients. In future studies, we expect to compare our template-guided minimally invasive technique with other minimally invasive TLIF approaches, which will help to provide a more comprehensive understanding of the advantages of this technique.

In conclusion, while it is crucial to account for the effects of skin movement and body position changes on the accuracy of screw placement, 3D-printed template navigation remains a novel and viable minimally invasive technique. This technology can effectively shorten operation time, reduce fluoroscopy duration and frequency, minimize trauma, and ensure precise screw placement. It helps prevent complications such as spinal cord and nerve injuries, as well as screw loosening due to repeated adjustments. For patients with severe lumbar degeneration, spinal stenosis, and unclear bony anatomical structures, this approach offers significant advantages and enhances surgical efficiency.

## Data Availability

The original contributions presented in the study are included in the article/Supplementary Material, further inquiries can be directed to the corresponding authors.
